# No survival benefit from adding chemotherapy to adjuvant radiation in advanced major salivary gland cancer

**DOI:** 10.1038/s41598-022-25468-9

**Published:** 2022-12-02

**Authors:** Nai-Wen Kang, Yu-Hsuan Kuo, Hung-Chang Wu, Chung-Han Ho, Yi-Chen Chen, Ching-Chieh Yang

**Affiliations:** 1 Division of Hematology and Oncology, Department of Internal Medicine, Chi Mei Medical Center, Tainan, Taiwan, ROC; 2 Department of Cosmetic Science, Chia-Nan University of Pharmacy and Science, Tainan, Taiwan, ROC; 3 Department of Hospital and Health Care Administration, Chia-Nan University of Pharmacy and Science, Tainan, Taiwan, ROC; 4 Department of Medical Research, Chi Mei Medical Center, Tainan, Taiwan, ROC; 5 Department of Information Management, Southern Taiwan University of Science and Technology, Tainan, Taiwan, ROC; 6 Department of Radiation Oncology, Chi Mei Medical Center, No. 901 Zhonghua Rd., Yung Kang District, Tainan City, 701 Taiwan, ROC; 7 Department of Pharmacy, Chia-Nan University of Pharmacy and Science, Tainan, Taiwan, ROC

**Keywords:** Cancer, Medical research, Oncology

## Abstract

This study aimed to compare survival of patients with advanced major salivary gland cancers treated with adjuvant chemoradiation therapy (CRT) versus radiotherapy (RT) alone, after surgical resection. The Taiwan Cancer Registry database was used to identify patients (2009–2017) with advanced (T3–4 or nodal positivity) major salivary gland cancers, treated post-surgically with adjuvant CRT or RT alone. Overall survival (OS) and disease-specific survival (DSS) evaluated using Kaplan–Meier. Stratified analyses conducted on clinicopathological features. A total of 395 patients were analyzed: 178 (45.1%) received adjuvant CRT; 217 (54.9%) received adjuvant RT alone. Median radiation dose was 66 Gy in 33 fractions. Cisplatin was most common chemotherapy regimen. After a median follow-up of 3.37 years, there was no significant difference in OS or DSS (*p* = 0.1354 and 0.3361, respectively) between groups. Adding chemotherapy to adjuvant RT was not significantly associated with improved OS (adjusted hazard ratio [aHR] 0.94; 95% CI 0.72–1.23) and DSS (aHR 0.96; 95% CI 0.72–1.28). Stratified analysis of clinicopathological features found no significant advantages for improved OS or DSS from adding chemotherapy to adjuvant RT. Thus, in this population database, the use of chemotherapy provided limited survival benefits in advanced major salivary gland cancers after surgical resection.

## Introduction

Salivary gland cancer is a rare tumor entity^1^. In Taiwan, they represent only 3% of all head and neck cancers and 0.3% of all malignant tumors^2^. Salivary glands consist of the three major salivary glands (parotid, submandibular, and sublingual) and the minor salivary glands. Molecular genetic studies have revealed a wide variety of histological subtypes of salivary gland cancers, including: adenoid cystic carcinoma (ACC); mucoepidermoid carcinoma (MEC); adenocarcinoma; salivary duct carcinoma, and; acinic cell carcinoma^3^.

The primary therapeutic approach for advanced salivary gland cancers is complete surgical resection, however, this can be challenging to perform due to invasion of adjacent structures. Therefore, adjuvant radiotherapy (RT) is indicated for advanced stage diseases and improves survival outcomes^4^. Several studies have identified high-risk features of recurrence in salivary gland cancers, including: advanced age; male; high grade; nodal positivity, and; positive margins^5–7^. Surgical resection followed by adjuvant RT results in excellent treatment outcomes with 5-year overall survival of approximately 70–80%, whereas the 5-year overall survival of locally advanced stage III and IV tumors ranges from 30 to 50%^8^. High incidences of local–regional recurrence and distant metastasis lead to a poorer prognosis in high-risk advanced salivary gland cancers and a more aggressive treatment strategy is warranted^9^. Compared to adjuvant RT alone, postoperative chemoradiotherapy (CRT) has been investigated in high-risk or locally advanced head and neck cancers^10,11^. The efficacy of adjuvant CRT after resection of salivary gland cancers currently lacks extensive prospective studies. There are some retrospective studies to assess the impact of adjuvant CRT in patients with resected salivary gland cancers. However, about the use of chemotherapy adding to adjuvant radiotherapy, some studies reported survival benefits from CRT while others did not^8,12–14^. In fact, most of these studies collected small sample sizes due to the rarity of this cancer type and insufficient information on important clinical features and treatment patterns, such as radiation dose, fields, and chemotherapy agents. Consequently, the treatment recommendation of adjuvant CRT remains controversial in advanced salivary gland cancers^15^.

The purpose of this study was to utilize the national Taiwan Cancer Registry (TCR) database to collect multi-institutional populations and compare the survival outcomes between adjuvant CRT and adjuvant RT alone, in patients with advanced major salivary gland cancers after complete surgical resection. Given the large sample size with a wide variety of histological subtypes and different histologies provided by the TCR database, a stratified analysis of various clinicopathological features could be performed in depth to identify the potential benefit of adjuvant CRT in high-risk patients.

## Materials and methods

### Ethics statement

This study was performed from our national database, which included no human subjects or personally identifying information and all data were analyzed anonymously. The Chi-Mei Medical Centre Institutional Review Board approved our study protocol and waived the need for individual retrospective consent. (IRB: CMFHR10801001). All methods were performed in accordance with relevant guidelines and regulations.

### Data source and patient population

From 2009 through 2017, patients with advanced major salivary gland cancer treated with curative surgery (pathological stage beyond T3 or N1), who are traditionally managed with CRT according to NCCN guidelines, were identified from the TCR and National Health Insurance (NHI) Research Database. The TCR database covered approximately all the cancer cases (97%) including the rare salivary gland cancer patients. In addition, TCR database have excellent accuracy than other database because the NHI institution reviewed charts to verify the data coding^2^.

**Figure1:**
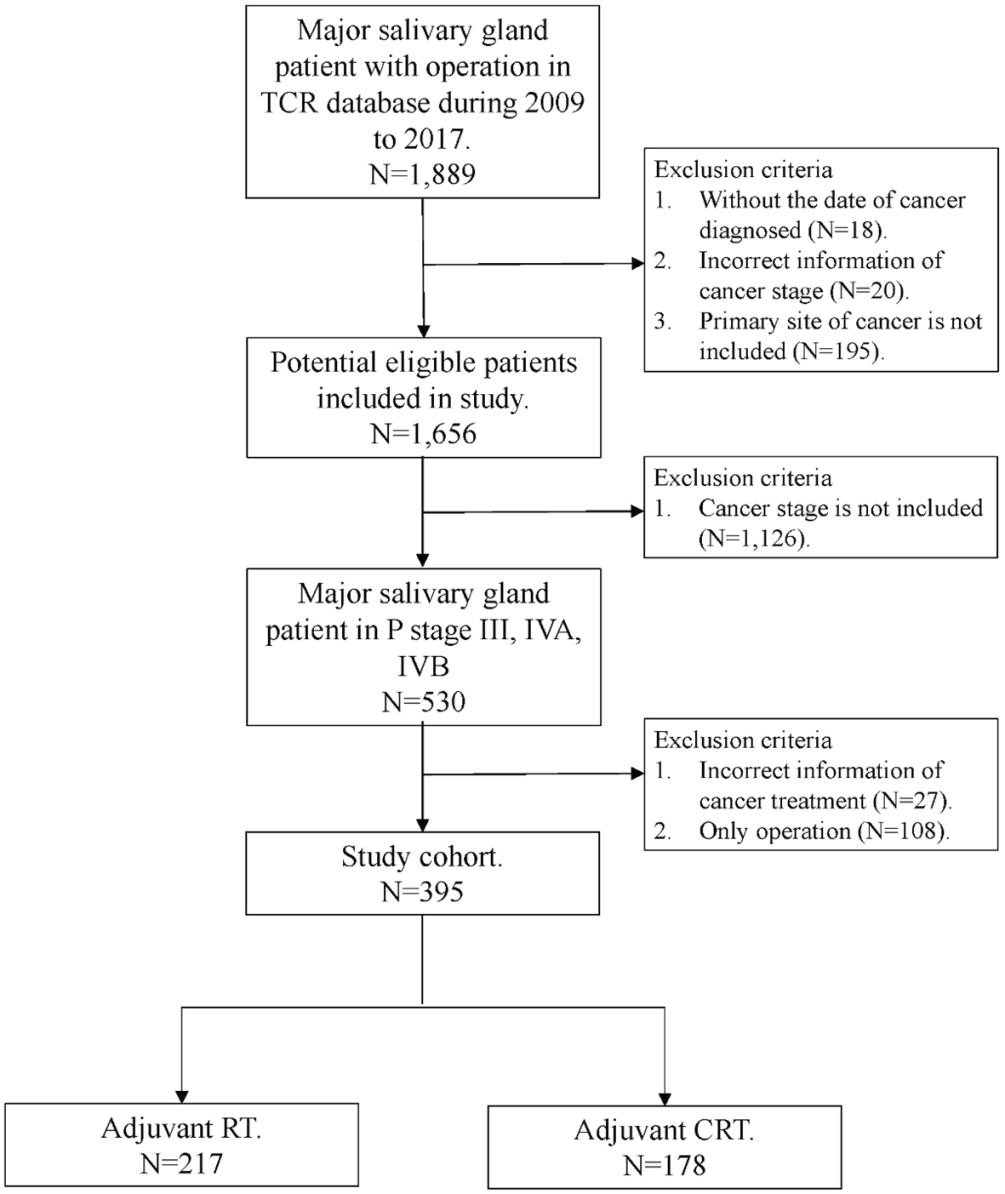
Flow chart.

Tumor location and histologic subtype were recorded according to the coding of *International Classification of Disease for Oncology, 3rd edition*. Tumor locations in the major salivary glands were categorized by: the parotid gland (C07.9); the submandibular gland (C0.80); the sublingual gland (C08.1); overlapping lesion of major salivary glands, and; major salivary gland, NOS (C0.88-0.89). Histologic subtypes were categorized as: adenocarcinoma (8140-7); adenoid cystic carcinoma (8200); mucoepidermoid carcinoma (8430); acinar cell carcinoma (8550-1), or; others (all other codes). Demographic and clinical data included: the date of diagnosis; site of the tumor; age; gender; margin status; histology grade; pathological tumor-node-metastasis (TNM) stage; chemotherapy; radiotherapy; cause of death, and; comorbidities. All patients were staged according to the American Joint Committee on Cancer classification system, 7th edition. Comorbid conditions were recorded based on the International Classification of Diseases, 9th Revision and were graded for severity using the Charlson Comorbidity Index (CCI)^16^. After excluding patients without surgical intervention or lacking complete data, a total of 395 advanced major salivary gland cancer patients treated with adjuvant RT and adjuvant CRT were included in this analysis. Figure 1 summarized the inclusion and exclusion criteria of our study population. The end points were overall survival (OS) and disease-specific survival (DSS).

### Statistical analysis

All statistical analyses were performed using SAS 9.4 for Windows (SAS Institute, Inc., Cary, NC, USA). Pearson’s chi-square test for categorical variables and the Wilcoxon ranked sum test for continuous variables were performed to estimate the efficacy of adjuvant RT versus CRT for advanced major salivary gland cancer patients. The OS and DSS rates were calculated using the Kaplan–Meier method, and the differences were compared using log-rank tests. Kaplan–Meier curves were plotted using STATA (version 12; Stata Corp., College Station, TX, USA). Risk was presented as hazard ratios (HRs) with 95% confidence intervals (CIs) and calculated using the Cox proportional hazard model after adjusting for other confounding variables. Stratified analyses were also performed based on clinicopathological features. *p* < 0.05 was considered significant.

## Results

### Patient characteristics

The demographic and clinicopathological characteristics of the patients in this study are summarized in Table 1. A total of 395 patients were identified, with 240 males (60.8%) and 155 females (39.2%). The median age (Q1–Q3) at diagnosis was 56 years old and the median follow‐up time (Q1-Q3) was 3.37 years. The most commonly used regimens were cisplatin (96.07%) followed by 5-fluorouracil (25.84%) and cyclophosphamide (6.74%) (Sup Table 1). The median (Q1-Q3) total dose of adjuvant radiotherapy was 66 Gy (range 60–70) in 33 fractions (range 32–35). Most patients received treatment using modern techniques, such as intensity modulated radiation therapy (IMRT) or volumetric modulated arc therapy (VMAT) (Sup Table 2). Among these patients, 217 (54.9%) received adjuvant RT and 178 (45.1%) received adjuvant CRT. In adjuvant CRT group, 154 patients (86.52%) received concurrent CRT (timeline of within 2 weeks). Patients who received adjuvant CRT were more likely to be younger (< 65 years old) and male and had a tumor with: poorly/undifferentiated grade; histologic subtype of adenoid cystic carcinoma, stage IVA and IVB; nodal positivity, or; positive margin status (all, *p* < 0.05).

**Table 1:** Patient and treatment characteristics of advanced major salivary gland cancer patients, n = 395.

Adjuvant treatment			
	RT	CRT	*P*-value
Overall	217	178	
**Age, year**			
Median (Q1–Q3)	57 (45–71)	56 (46–62)	0.1020
< 65	143 (65.90)	147 (82.58)	0.0002
≧65	74 (34.10)	31 (17.42)	
**Gender**			
Male	115 (53.00)	125 (70.22)	0.0005
Female	102 (47.00)	53 (29.78)	
**Grade**			
Well/moderately	172 (79.26)	102 (57.30)	< 0.0001
Poorly/undifferentiated	45 (20.74)	76 (42.70)	
**Primary site criteria**			
Parotid gland	148 (68.20)	123 (69.10)	0.1220
Submandibular gland	50 (23.04)	47 (26.40)	
Sublingual gland	13 (5.99)	8 (4.49)	
Overlapping lesion of major salivary glands and major salivary gland, NOS	6 (2.76)	0 (0.00)	
**Histological types**			
Adenoid cystic carcinoma	61 (28.11)	25 (14.04)	0.0008
Others	156 (71.89)	153 (85.96)	
**pTNM stage**			
III	139 (64.06)	56 (31.46)	< 0.0001
IVA	78 (35.94)	116 (65.17)	
IVB	0 (0.00)	6 (3.37)	
**pT classification**			
1–2	21 (9.68)	47 (26.40)	< .0001
3–4	196 (90.32)	131 (73.60)	
**pN classification**			
0	142 (65.44)	54 (30.34)	< 0.0001
1–3	75 (34.56)	124 (69.66)	
**Margin**			
Negative	119 (54.84)	71 (39.89)	0.0215
Positive	82 (37.79)	90 (50.56)	
Unknown	16 (7.37)	17 (9.88)	
**CCI**			
0	141 (64.98)	116 (65.17)	0.6127
1	32 (14.75)	21 (11.80)	
≧2	44 (20.28)	41 (23.03)	
**Follow-up period, years**			
Median (Q1–Q3)	3.41 (1.83–6.11)	3.33 (1.58–5.67)	0.3436

*RT* radiotherapy, *CRT* chemoradiotherapy, *CCI* Charlson Comorbidity Index.

*P*-value was calculated from Pearson’s Chi-square for categorical variables and Wilcoxon rank sum test was used to comparing the medians between the two groups.

### Survival analysis

**Figure 2:**
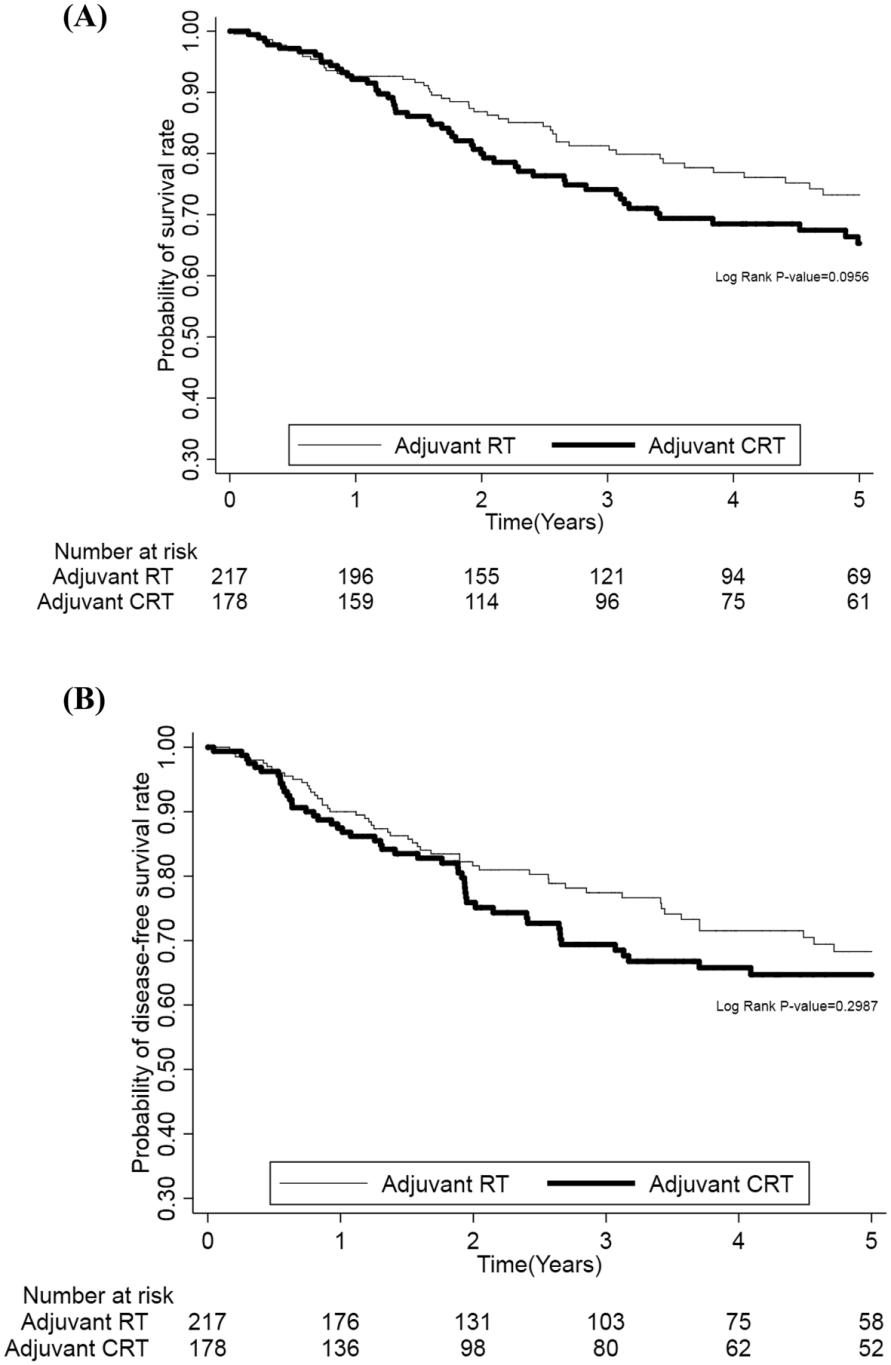
Kaplan–Meier plots of (**A**) overall survival and (**B**) disease-free survival in advanced major salivary gland cancer patients receiving chemoradiotherapy and radiotherapy alone.

OS and DSS of resected advanced major salivary gland cancers treated with adjuvant RT or CRT were compared using the Kaplan–Meier method. As presented in Fig. 2, there were no significant differences in OS and DSS between the patients who received adjuvant RT and those who received adjuvant CRT (*p* = 0.1354 and 0.3361, respectively). After adjustment for confounders (Tables 2, 3), multivariable analysis indicated that adding chemotherapy to adjuvant RT was not significantly associated with better OS (adjusted hazard ratio [aHR] 0.94; 95% CI 0.72–1.23) and DSS (aHR 0.96; 95% CI 0.72–1.28). In order to further differentiate the effect of adjuvant CRT, we performed stratified analysis for OS and DSS according to various clinicopathological features. Table 4 shows there were no significant advantages from the addition of chemotherapy to adjuvant RT resulting in improved OS or DSS.

**Table 2:** Risk factor of overall survival in advanced major salivary gland cancer patients, n = 395.

	Crude HR (95% C.I.)	*P*-value	Adjusted HR (95% C.I.)	*P*-value
CRT vs RT	0.90 (0.72–1.14)	0.3771	0.94 (0.72–1.23)	0.6654
**Age**				
< 65	Ref.		Ref.	
≧65	1.33 (1.01–1.75)	0.0434	1.36 (1.01–1.83)	0.0410
**Gender**				
Male	0.98 (0.78–1.23)	0.8538	0.99 (0.78–1.26)	0.9320
Female	Ref.		Ref.	
**Grade**				
Well/moderately	Ref.		Ref.	
Poorly/undifferentiated	1.15 (0.89–1.48)	0.2912	1.14 (0.87–1.50)	0.3296
**Primary site criteria**				
Parotid gland	Ref.		Ref.	
Submandibular gland	0.97 (0.74–1.27)	0.8388	1.02 (0.76–1.36)	0.9152
Sublingual gland	1.19 (0.73–1.96)	0.4830	1.38 (0.80–2.38)	0.2511
Overlapping lesion of major salivary glands and major salivary gland, NOS	1.02 (0.42–2.49)	0.9578	1.01 (0.40–2.52)	0.9850
**Histological types**				
Adenoid cystic carcinoma	0.87 (0.66–1.14)	0.3013	0.79 (0.58–1.10)	0.1622
Others	Ref.		Ref.	
**pT classification**				
1–2	Ref.		Ref.	
3–4	1.05 (0.78–1.41)	0.7723	0.95 (0.66–1.36)	0.7653
**pN classification**				
0	Ref.		Ref.	
1–3	0.90 (0.72–1.13)	0.3565	0.84 (0.63–1.11)	0.2204
**Margin**				
Positive	0.89 (0.70–1.13)	0.3508	0.91 (0.70–1.18)	0.4668
Negative	Ref.		Ref.	
**CCI**				
0	Ref.		Ref.	
1	0.78 (0.56–1.09)	0.1493	0.77 (0.54–1.08)	0.1308
≧2	0.89 (0.66–1.20)	0.4353	0.84 (0.62–1.14)	0.2635

*RT* radiotherapy, *CRT* chemoradiotherapy, *CCI* Charlson Comorbidity Index.

**Table 3:** Risk factor of disease-free survival in advanced major salivary gland cancer patients, n = 395.

	Crude HR (95% C.I.)	*P*-value	Adjusted HR (95% C.I.)	*P*-value
CRT vs RT	0.89 (0.70–1.14)	0.3651	0.96 (0.72–1.28)	0.7557
**Age**				
< 65	Ref.		Ref.	
≧65	1.30 (0.96–1.76)	0.0953	1.35 (0.97–1.88)	0.0798
**Gender**				
Male	0.98 (0.77–1.26)	0.8858	1.01 (0.79–1.30)	0.9296
Female	Ref.		Ref.	
**Grade**				
Well/moderately	Ref.		Ref.	
Poorly/ undifferentiated	1.12 (0.85–1.46)	0.4299	1.13 (0.84–1.50)	0.4222
**Primary site criteria**				
Parotid gland	Ref.		Ref.	
Submandibular gland	0.93 (0.69–1.24)	0.6065	0.98 (0.72–1.33)	0.8780
Sublingual gland	1.13 (0.65–1.94)	0.6665	1.31 (0.72–2.38)	0.3708
Overlapping lesion of major salivary glands and major salivary gland, NOS	0.94 (0.30–2.93)	0.9098	0.95 (0.29–3.04)	0.9249
**Histological types**				
Adenoid cystic carcinoma	0.86 (0.64–1.16)	0.3328	0.77 (0.54–1.09)	0.1424
Others	Ref.		Ref.	
**pT classification**				
1–2	Ref.		Ref.	
3–4	1.07 (0.78–1.48)	0.6625	0.97 (0.65–1.43)	0.8618
**pN classification**				
0	Ref.		Ref.	
1–3	0.89 (0.70–1.14)	0.3598	0.84 (0.62–1.14)	0.2621
**Margin**				
Positive	0.90 (0.70–1.17)	0.4368	0.93 (0.71–1.23)	0.6104
Negative	Ref.		Ref.	
**CCI**				
0	Ref.		Ref.	
1	0.83 (0.57–1.21)	0.3322	0.83 (0.56–1.21)	0.3265
≧2	0.89 (0.65–1.21)	0.4430	0.84 (0.60–1.17)	0.2879

*RT* radiotherapy, *CRT* chemoradiotherapy, *CCI* Charlson Comorbidity Index.

**Table 4:** Stratified analysis of chemotherapy use or not for the overall survival and disease-free survival according to different clinicopathological features, n = 395.

Subgroup variable	Overall survival		Disease-free survival	
	Crude HR (95% C.I.)	Adjusted HR^a^ (95% C.I.)	Crude HR (95% C.I.)	Adjusted HR^b^ (95% C.I.)
**Age**				
< 65	0.93 (0.72–1.20)	0.96 (0.72–1.30)	0.91 (0.69–1.19)	0.96 (0.70–1.32)
≧65	0.95 (0.56–1.60)	0.84 (0.42–1.67)	1.00 (0.54–1.84)	1.08 (0.42–2.76)
**Gender**				
Male	0.89 (0.66–1.20)	0.91 (0.64–1.28)	0.89 (0.65–1.22)	0.97 (0.66–1.42)
Female	0.91 (0.63–1.34)	1.10 (0.71–1.69)	0.89 (0.59–1.34)	1.04 (0.65–1.65)
**Grade**				
Well/moderately	0.85 (0.65–1.13)	0.95 (0.69–1.30)	0.87 (0.65–1.17)	0.93 (0.66–1.32)
Poorly/undifferentiated	0.95 (0.60–1.48)	1.07 (0.64–1.79)	0.87 (0.54–1.39)	1.02 (0.59–1.76)
**Primary site criteria**				
Parotid gland	0.99 (0.75–1.30)	1.04 (0.76–1.42)	0.98 (0.73–1.31)	1.05 (0.75–1.47)
Submandibular gland	0.76 (0.47–1.20)	0.92 (0.50–1.69)	0.74 (0.45–1.23)	0.93 (0.48–1.81)
Sublingual gland	0.66 (0.24–1.79)	0.17 (0.03–1.05)	0.61 (0.20–1.88)	–
Overlapping lesion of major salivary glands and major salivary gland, NOS	–	–	–	–
**Histological types**				
Adenoid cystic carcinoma	0.84 (0.50–1.41)	0.79 (0.42–1.46)	0.85 (0.48–1.50)	0.83 (0.42–1.65)
Others	0.89 (0.69–1.16)	1.00 (0.74–1.35)	0.88 (0.67–1.16)	1.00 (0.72–1.39)
**pT stage**				
T1–2	1.14 (0.64–2.03)	1.13 (0.58–2.21)	0.99 (0.52–1.88)	0.95 (0.44–2.04)
T3–4	0.86 (0.66–1.12)	0.92 (0.68–1.24)	0.88 (0.67–1.17)	0.96 (0.70–1.33)
**pN stage**				
N0	0.81 (0.57–1.15)	0.76 (0.52–1.13)	0.83 (0.57–1.20)	0.80 (0.53–1.23)
N1–3	1.06 (0.75–1.51)	1.15 (0.77–1.73)	1.03 (0.70–1.51)	1.09 (0.69–1.71)
**Margin**				
Positive	0.83 (0.59–1.19)	0.73 (0.49–1.10)	0.75 (0.51–1.10)	0.66 (0.42–1.02)
Negative	0.96 (0.69–1.35)	1.20 (0.80–1.80)	1.02 (0.71–1.47)	1.31 (0.84–2.05)
**CCI**				
0	0.87 (0.66–1.16)	0.98 (0.70–1.38)	0.84 (0.62–1.13)	0.95 (0.66–1.37)
1	0.91 (0.48–1.72)	0.74 (0.29–1.92)	0.95 (0.47–1.93)	1.45 (0.39–5.40)
≧2	1.00 (0.59–1.69)	1.00 (0.56–1.79)	1.07 (0.62–1.87)	0.95 (0.51–1.75)

*RT* radiotherapy, *CRT* chemoradiotherapy, *CCI* Charlson Comorbidity Index.

^a^Adjusted model of overall survival between CRT and RT alone was shown as Table 3.

^b^Adjusted model of cancer-free survival between CRT and RT alone was shown as Table 4.

## Discussion

Previous studies have investigated the effects of adding chemotherapy to adjuvant RT to address poor prognostic factors, such as elderly, male, high grade, nodal involvement, or positive margins^6,7,17^. This analysis, using a nationwide cancer registry database, found that there were no significant differences in OS or DSS between adjuvant CRT, and RT alone, in patients with operated advanced major salivary gland cancers, even for those with unfavorable prognostic factors. These findings could help guide treatment management decisions. Future studies will be needed to evaluate the efficacy of various chemotherapy regimens for treatment of resected advanced major salivary gland cancers.

This study appears to be one of the larger population-based studies to identify the benefit of adding chemotherapy to adjuvant RT in advanced salivary gland cancers. There were 395 patients in which advanced major salivary gland cancers were identified from the TCR database, from 2009 through 2017. The TCR database was implemented in 1979. In order to better evaluate cancer treatment patterns and survival outcomes, the TCR database has been revised since 2002 to include both the stage at the time of diagnosis and the course of treatment. It is an informative nationwide database for academic research with 97% completeness^2^. Comparing to previous studies, our sample size was relatively large to perform an in-depth assessment of the impact of chemotherapy on survival. Moreover, TCR database had comprehensive tumor and treatment records including a wide variety of histological subtypes and different histologies, radiotherapy, and specific chemotherapy agents. It worth to be mentioned that previously published retrospective studies frequently pooled both patients with early and advanced stages^12,13,18–20^. This study focused on advanced stage patients after curative surgical resection.

Due to the rarity of salivary gland cancers there are no randomized studies, and a paucity of prospective studies, that attempt to identify the benefit of adjuvant CRT after resected salivary gland cancers. The rationale of the addition of chemotherapy to adjuvant RT is extrapolated from head and neck squamous cell carcinoma treatment trials^10,11^. From a practical perspective, the current evidence for the use of adjuvant CRT in salivary gland cancers remains contradictory. Certain single institution experiences suggest that adjuvant CRT appeared to be effective and resulted in excellent local control rates in advanced stage or high-risk patients^13,21^. However, the sample sizes in these studies were relatively small and the statistical power reflected this limitation. Conversely, in a retrospective analysis using the National Cancer Database, Amini et al. demonstrated no improvement of OS with the addition of chemotherapy to adjuvant RT in patients with salivary gland cancers^8^. Some retrospective studies also failed to demonstrate survival benefits with the use of adjuvant CRT after resection of salivary gland cancers^19,20^. In concordance with these larger-scale retrospective studies, our study demonstrated no significant survival benefits of adjuvant CRT in advanced salivary gland cancers upon univariate and multivariate analyses.

Amini et al. conducted a retrospective analysis from the National Cancer Data Base and identified lower OS with adjuvant CRT compared with RT alone in salivary gland cancers on multivariate analysis^8^. A total of 2210 patients with high-risk major salivary gland cancers with grade 2 to 3 disease and one of the following adverse features: T3–T4 stage; N1–N3 stage, or; positive margins. Mifsud et al. demonstrated that adjuvant CRT was associated with lower OS and progression-free survival in a retrospective study of 140 patients with high-risk major or minor salivary gland cancers^20^. Gebhardt et al. performed a retrospective series of 128 patients with salivary gland cancers in the UPMC Cancer Center network, and a subset analysis of 54 patients with at least one high-risk factor, including: stage T3–T4, lymph node involvement; close-to node involvement (≤ 1 mm), or; positive surgical resection margins^19^. No survival benefits were seen with the use of adjuvant CRT in both the overall study population and the subset of 54 with high-risk factors. Similarly, our analysis of 395 Asian patients with advanced stage (T3–T4 or nodal positivity) major salivary gland cancers from the national TCR database demonstrated no improvement in OS or DSS following the addition of chemotherapy to adjuvant RT. Following-up on the suspicion that more toxic effects related to the addition of chemotherapy to adjuvant RT may increase risk of mortality and contribute to negative outcomes, an analysis from Surveillance, Epidemiology, and End Results database suggested increased toxicity and mortality with combined treatment modality of adjuvant CRT^14^. Currently, two ongoing randomized phase II/III trials, NCT01220583 (RTOG 1008) and NCT02998385, are the first to compare adjuvant CRT versus RT alone in patients with high-risk salivary gland cancers after radical resection The eligibility criteria of these trials include T3–4, N1–3, or T1–2 with close or positive surgical margin. These prospective randomized trials are designed to solve this challenging problem. Additionally, NCT02776163 collected patients with advanced stage or high-risk salivary gland cancers to evaluate the efficacy and safety of chemoradiation with cisplatin plus docetaxel or cisplatin alone, in an attempt to find a better radiosensitizer to improve the treatment outcome of this rare and heterogenous cancer type.

For the patients who received adjuvant CRT in this study of 395 patients, most were treated with the cisplatin-based regimen. Cisplatin is a well-known radiosensitizer for a variety of cancers, with the mechanism of DNA double-strand break repair interference^18^. To date, several chemotherapeutic agents have been proven to be effective against salivary gland cancers, including carboplatin, paclitaxel, 5-fluorouracil, cyclophosphamide, and vinorelbine^13,22,23^. Schoenfeld et al. conducted a retrospective study of 35 salivary gland cancers that suggested excellent local control of adjuvant CRT in patients with adverse prognostic factors^13^. The most commonly used chemotherapy regimen in Schoenfeld was carboplatin and paclitaxel. Katori et al. used a CRT regimen of cyclophosphamide, pirarubicin, and cisplatin for 17 locally advanced salivary gland cancers^22^. The regimen of Katori et al. yielded excellent antitumor activity. It remains unclear whether cisplatin is the most appropriate radiosensitizer for salivary gland cancers. Furthermore, the choice of chemotherapeutic agents used as radiosensitizer may be based on histologic subtypes, according to the French Network of Rare Head and Neck Tumors^24^. Patients with ACC are reportedly sensitive to carboplatin and paclitaxel-based CRT regimen^25^. Patients with MEC may be responsive to cisplatin and 5-FU^23^.

Recent advances in molecular genomics have developed a potential role of targeted therapy, particularly against epidermal growth factor receptor and human epidermal growth factor receptor 2 (HER2). For patients who are ineligible for cisplatin with CRT for locally advanced head and neck squamous cell carcinoma, concurrent cetuximab is an alternative option^10^. HER2 positivity has been found in some advanced salivary gland cancers. A phase II study, Haddad et al., identified one case of long-lasting partial response treated with trastuzumab, a HER2-targeted therapy^26^. Moreover, immunotherapy is increasingly becoming a treatment option for patients with metastatic salivary gland tumors. Pembrolizumab, an immune checkpoint inhibitor, is a treatment option for patients with previously treated high microsatellite instability metastatic salivary gland cancers, based on the result from the KEYNOTE 158 trial^27^. In addition to conventional chemotherapeutic agents, current studies aimed at defining the optimal radiosensitizer for targeted therapy or immunotherapy are warranted. Combining molecular-driven targeted therapy or immunotherapy with RT may be another way to improve outcomes in high-risk advanced salivary gland cancers.

There were certain limitations in this study. First, although most of the important adverse prognostic factors were analyzed, some risk factor for outcome were unavailable such as perineural and angiolymphatic invasion and performance status (ECOG) which may influence the use of adjuvant RT or CRT^28,29^. Secondly, salivary gland cancers are heterogenous with variable survival outcomes among different histologic subtypes^30^. Our study included all of tumor histologic subtypes, which made the evaluation of treatment outcome more complicated. Thirdly, the administration of chemotherapy without dose adjustment or interruption is difficult to execute because of unacceptable treatment-related toxicity. Information regarding cycles of chemotherapy and treatment-related toxicity was not available in the TCR database. We were unable to assess the compliance to the use of chemotherapy. Finally, the 5-year overall survival of advanced salivary gland cancers is approximately 70%, whereas the 5-year overall survival of locally advanced stage III and IV tumors ranges from 30 to 50%^8^. The median follow-up period of our study was 3.37 years, while sufficient, a longer follow-up period would be accountable for any late relapse.

In conclusion, the findings of this study indicated that there are no survival benefits gained from adding chemotherapy to adjuvant RT after the resection of advanced major salivary gland cancers. Future prospective randomized trials would be needed in order to confirm the efficacy of adjuvant CRT. More studies investigating novel agents based on molecular genetic discoveries will promote personalized approaches and improve the treatment outcome for this rare and histologically diverse disease.

## Supplementary Information

Supplementary Information.

## Data Availability

The data presented in current study are available on request from the corresponding author.
