# Supplementary material for: No survival benefit from adding chemotherapy to adjuvant radiation in advanced major salivary gland cancer
Source: Sci Rep. 2022 Dec 2;12:20862. doi: 10.1038/s41598-022-25468-9 (PMC9718855; doi:10.1038/s41598-022-25468-9)
Supplement: Supplementary file 1 — Supplementary Information. [file 41598_2022_25468_MOESM1_ESM.docx]

**Supplementary table 1.** **Chemotherapy regimens use information, N=178**

| **Regimens** | **N** | **%** |
| --- | --- | --- |
| Cisplatin | 171 | 96.07 |
| 5-FU | 46 | 25.84 |
| Cyclophosphamide | 12 | 6.74 |
| Carboplatin | 9 | 5.23 |
| Docetaxel | 3 | 1.74 |
| Methotrexate | 3 | 1.74 |
| Cetuximab | 2 | 1.12 |

**Supplementary table 2.** Radiation treatment parameters

|  | **RT** | **CRT** |
| --- | --- | --- |
| **Overall** | 217 | 178 |
| **OP-RT interval, days** |  |  |
| Median (Q1-Q3) | 35 (27-42) | 34.5 (28-42) |
| **RT treatment time, days** |  |  |
| Median (Q1-Q3) | 46 (44-49) | 47 (44-50) |
| **Number of Fractions** |  |  |
| Median (Q1-Q3) | 33 (32-33) | 33 (33-35) |
| **RT dose, Gy** |  |  |
| Median (Q1-Q3) | 66 (60-66) | 66 (66-70) |
| Min-Max | 1.8-76.3 | 16-73.5 |
| **RT techniques, missing (N=45)** |  |  |
| 2D/3DCRT | 12 | 0 |
| IMRT | 138 | 114 |
| VMAT | 43 | 44 |

Abbreviations: OP, operation; RT, radiotherapy; IMRT, intensity modulated radiation therapy; VMAT, volumetric modulated arc therapy.
